# P05.46. Spiritual needs of veterans: healthcare implications for returning troops

**DOI:** 10.1186/1472-6882-12-S1-P406

**Published:** 2012-06-12

**Authors:** B Chang, N Stein, M Stewart, A Hendricks, L Skarf

**Affiliations:** 1 VA Boston Healthcare System, Boston, Massachusetts, USA

## Purpose

Spirituality plays an important role for many people. In particular, its crucial role among people at the end-of-life (EoL) has been recognized. Currently one out of 4 deaths in the US is a veteran. The study of spirituality among veterans who are at the EoL is therefore urgently needed. The objectives of this study are to understand the spiritual needs and spiritual care provided to veterans at the EoL in the Veteran Administration (VA) Healthcare System. We particularly focused on how military experience impacts spirituality and the spiritual care for veterans who, at the EoL, are still suffering from these experiences.

## Methods

We conducted a qualitative study that interviewed VA chaplains, veterans who are at the EoL and their families. The interviews were recorded, transcribed, and then analyzed based on Grounded Theory.

## Results

Veterans and their families expressed a range of spiritual needs including religious activities, divine intervention (e.g., God answer prayers), reconnection to their religion, time with family, compassion/love, respect, and conversations about spiritual concerns. One unique need of veterans is to process negative impacts (e.g., guilt and anger) from events that occurred during their combat experience. Chaplains reported various approaches for addressing this special need. Some veterans indicated the desires for more frequent and longer visits from chaplains. Veterans however reported that spiritual care can be provided by professionals (e.g., doctors and nurses) other than chaplains.

## Conclusion

The results of our study highlight the importance of addressing spiritual needs of veterans in healthcare. The study finding that some veterans, who at the EoL, are still struggling with spiritual issues related to events that occurred during combat many decades earlier indicates early interventions for addressing these issues might prevent the long-term suffering of more recent veterans, such as the recently returned troops from Iraq.

